# Weaponizing volatiles to inhibit competitor biofilms from a distance

**DOI:** 10.1038/s41522-020-00174-4

**Published:** 2021-01-05

**Authors:** Qihui Hou, Alona Keren-Paz, Elisa Korenblum, Rela Oved, Sergey Malitsky, Ilana Kolodkin-Gal

**Affiliations:** 1 Department of Molecular Genetics, Weizmann Institute of Science, Rehovot, Israel; 2 Department of Plant and Environmental Sciences, Weizmann Institute of Science, Rehovot, Israel; 3 Metabolic Profiling Unit, Weizmann Institute of Science, Rehovot, Israel

**Keywords:** Microbial communities, Biofilms

## Abstract

The soil bacterium *Bacillus subtilis* forms beneficial biofilms that induce plant defences and prevent the growth of pathogens. It is naturally found in the rhizosphere, where microorganisms coexist in an extremely competitive environment, and thus have evolved a diverse arsenal of defence mechanisms. In this work, we found that volatile compounds produced by *B. subtilis* biofilms inhibited the development of competing biofilm colonies, by reducing extracellular matrix gene expression, both within and across species. This effect was dose-dependent, with the structural defects becoming more pronounced as the number of volatile-producing colonies increased. This inhibition was mostly mediated by organic volatiles, and we identified the active molecules as 3-methyl-1-butanol and 1-butanol. Similar results were obtained with biofilms formed by phylogenetically distinct bacterium sharing the same niche, *Escherichia coli*, which produced the biofilm-inhibiting 3-methyl-1-butanol and 2-nonanon. The ability of established biofilms to inhibit the development and spreading of new biofilms from afar might be a general mechanism utilized by bacterial biofilms to protect an occupied niche from the invasion of competing bacteria.

## Introduction

In nature, bacteria form complex and differentiated multicellular communities, known as biofilms^1^. The coordinated actions of many cells, communicating and dividing labour, improve the ability of the biofilm community to resist antibiotics and environmental assaults^2–4^. Bacterial biofilms are associated with persistent chronic infections, and thus pose a global threat of extreme clinical importance^5,6^. However, in many instances, biofilms can be beneficial. One example is the biocontrol agents that form biofilms on the surface of plant roots, producing antibiotics that prevent the growth of bacterial and fungal pathogens and inducing the plant systemic response^7–10^.

The Gram-positive bacterium *Bacillus subtilis* is a genetically manipulatable model organism for biofilm development and for beneficial environmental activities of bacteria^11^. The main organic components of its biofilm extracellular matrix (ECM) are (i) exopolysaccharides (EPS), synthesised by the *epsA-O* operon-encoded genes; (ii) BslA, a protein forming a hydrophobic coat protecting the biofilm^12^; and (iii) the amyloid-like protein TasA, encoded in the three-gene operon *tapA-sipW-tasA*^13^. Amyloid-like proteins such as TasA are extremely common in bacterial biofilms, and their assembly into fibres is important for the integrity and structure of biofilms^14^. In addition to its structural role, the ECM is essential for *B. subtilis* spreading^8,15,16^.

Biofilm formation is initiated by a signalling cascade that simultaneously inhibits motility and activates ECM expression. In *B. subtilis*, phosphorylation of the master-regulator Spo0A activates SinI, which in turn neutralises the SinR repressor, therefore allowing the production of ECM components (EPS and TasA) and repressing the expression of *hag* (encoding flagellin)^17–19^. In addition, Spo0A neutralises AbrB repressor, therefore releasing the inhibition of *bslA* expression. In a parallel cascade, *bslA* expression is also activated by the sensor kinase DegS and the response regulator DegU^20,21^. ECM expression is necessary for the development of a highly organised 3D architecture, and the precise spatial organisation that results in a complex differentiated community. Therefore, mutants in *spo0A* and *degU* fail to develop the characteristic biofilm structure, remaining featureless. This correlation between ECM expression, colony structure and biofilm development has been reported for both Gram-positive and Gram-negative bacteria^22–24^.

Bacterial biofilm colonies growing on solid medium offer a controlled and reproducible experimental system that has facilitated the discovery of the molecular pathways governing biofilm development. In a similar approach, assessing intra-species interactions between biofilm colonies growing on agar plates has been previously utilised to study molecular ecology, uncovering the genetic circuits responsible for complex bacterial behaviour^25–31^.

Like other bacteria, *B. subtilis* produces a wide repertoire of volatile compounds (VCs)—biologically active airborne molecules^32^. VCs are used by bacteria to interact with their environment, and were first identified as cross-kingdom signals influencing survival and behaviour of fungi, plants and vertebrates^33–35^. However, VCs are also used as chemical signals during bacteria–bacteria interactions, altering motility, growth and differentiation, affecting virulence and boosting antibiotic and stress resistance of various bacterial species^36^.

Recent evidence suggests that VCs may also modulate the development of bacterial communities. In nature, biofilms exist in an extremely competitive environment, and thus engage in both positive and negative interaction. While the ability to coordinate biofilm development within a community is beneficial in some cases; the ability to inhibit competing biofilm development is no less significant. In a systematic study of biological activity of VCs on four bacterial species, several VCs (including 1-butanol, ethanol, indole and others) were found to affect biofilm formation as judged by bacterial adhesion to a microtiter plate^37^, but the effects were highly compound- and species-specific. For *B. subtilis*, it has been reported that ammonia^38^ and acetic acid^39^ produced by *B. subtilis* pellicles (floating biofilms) stimulate neighbouring pellicle formation. On the other hand, one study has shown that biocontrol strain *Bacillus amyloliquefaciens* SQR-9 produced volatiles inhibiting the growth of plant pathogen *Ralstonia solanacearum*. In addition to the effect of VCs on the growth of the pathogen, the VCs also reduced colony spreading, motility, production of exopolysaccharides and surface attachment of their own producers^40^. Those results suggest that in nature, the role of VCs is highly context-dependent, and that additional studies are needed to understand the mechanisms mediating the effects of VCs produced by biofilms during ecological microbial interactions.

We here explored the dose-dependent activity of VCs in inter- and intra-species interaction between biofilms, and found when bacterial communities reach critical biomass, they can use VCs as a specific regulatory signal to inhibit biofilm development of potential competitors. Biomass-dependent inhibition of neighbouring biofilms by VCs was conserved in *B. subtilis* and *Escherichia coli*. We found that this inhibition was mediated by dysregulation of biofilm transcription programme—and that the expression of genes encoding the ECM components was inhibited by specific VCs produced by biofilms.

## Results

### Volatiles can inhibit biofilm development from a distance

**Fig. 1:**
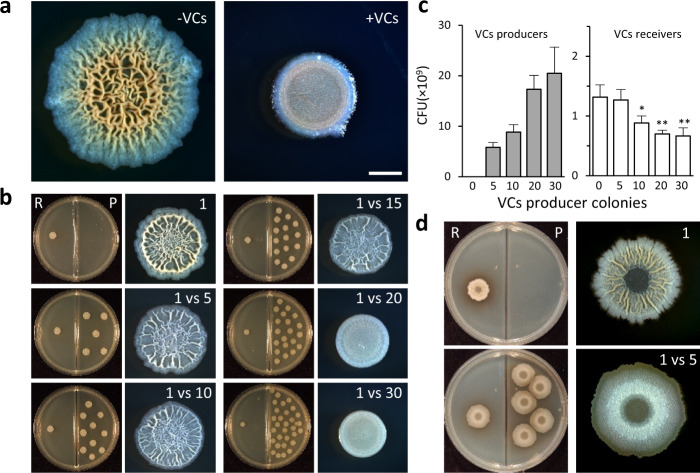
VCs produced by *B. subtilis* biofilms inhibit biofilm development in a dose-dependent manner. **a** Top-down images of *B. subtilis* 3610 biofilm colonies, grown on solid B4 medium, either alone (−VCs) or in the presence of volatile compounds produced by 80 neighbouring colonies (+VCs). Colonies were incubated for 4 days at 30 °C. Scale bar 2 mm. Images are representative of (*n* > 3) independent experiments. **b** *B. subtilis* 3610 biofilm colonies (R—receiver) grown in divided Petri dishes on solid B4 medium in the presence of an indicated number of neighbouring colonies (P—producer). Left—experimental setting, right—a close-up of the receiving colony. Colonies were incubated for 2 days at 30 °C. Images are representative of (*n* > 3) independent experiments. **c** Colony-forming units were determined for VCs producer (left) and receiver (right). Colonies (*n* = 6) were grown as in **b**. *P*-values, as determined by ANOVA followed by Tukey HSD are indicated (*pVal < 0.01, **pVal < 0.001 vs 0 VCs producers). **d** *B. subtilis* 3610 biofilm colonies (R—receiver) grown on solid MSgg medium in the presence of 5 neighbouring colonies (P—producer). Left—experimental setting, right—a close-up of the receiving colony. Colonies were incubated for 2 days at 30 °C. Images are representative of (*n* > 3) independent experiments.

We first tested the effect of VCs produced by *B. subtilis* biofilm colonies on the development of neighbouring colonies. Towards this goal, colonies were grown on solid rich biofilm-inducing medium physically separated but sharing headspace (Supplementary Fig. 1^41^). In the presence of VCs, the development of neighbouring biofilms was inhibited, and the colonies formed were small and flat (Fig. 1a). This effect was gradual and dose-dependent, with the reduced size and structural defects becoming more pronounced as the number of producing colonies increased (Fig. 1b). Severe defects in colony morphology could be observed only once a critical mass of VCs producers was achieved—at least 20 colonies were required to completely prevent the formation of the characteristic 3D colony architecture (Fig. 1b). With time, this inhibition was slightly relieved, however, biofilms grown in the presence of VCs producers remained smaller and less developed (Supplementary Fig. 2). CFU analysis revealed that the defective biofilm structure was associated with reduced cell number—as the total number of cells producing VCs increased, the number of cells in each colony declined (Fig. 1c).

This inhibition of biofilm development was robust and not medium-dependant, as it was also observed in the defined biofilm-inducing medium MSgg (Fig. 1d). Colonies formed on MSgg are larger and contain more cells—and consistently, less colonies were needed to reach the biomass critical for inhibition of biofilm development. Similar results were observed when the optimal nitrogen source (amino acids) was replaced with plant root exudate (Supplementary Fig. 3), to better mimic the conditions present in the rhizosphere.

### Volatiles can specifically dysregulate the biofilm developmental programme

**Fig. 2:**
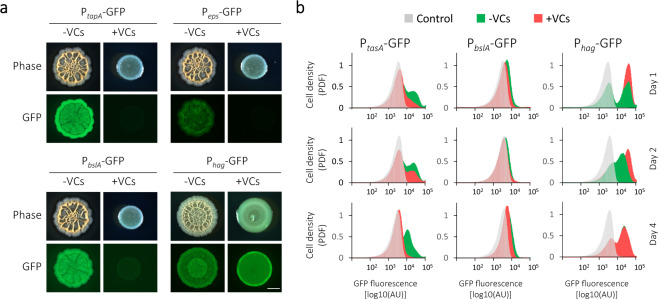
Volatiles produced by *B. subtilis* biofilms inhibit ECM expression. **a** Top-down phase and fluorescent (GFP) images of *B. subtilis* 3610 strains carrying P_*eps*_-GFP, P_*bslA*_-GFP, P_*tapA*_-GFP or P_*hag*_-GFP were incubated either alone (−VCs) or in the presence of 20 volatile producers (+VCs). Colonies were inoculated on solid B4 medium, and incubated for 2 days at 30 °C. Scale bar 2 mm. Images are representative of (*n* > 3) independent experiments. **b** Flow cytometry analysis of colonies grown as in **a** for 1, 2 or 4 days, as indicated. Colonies were grown either alone (green, −VCs) or in the presence of 20 volatile producers (red, +VCs). Control (grey)—autofluorescence levels of the parental non-fluorescent strain. Shown are representative results of three independent experiments performed with least two technical repeats.

Biofilm development requires precise regulation of specific molecular pathways, such as the coordinated production of several ECM components. We, therefore, set to test whether the phenotypic defect in biofilm structure reflects specific dysregulation of biofilm developmental programme. We utilised promoter-fusion reporters for ECM and motility genes to examine the effect of VCs on their expression. The expression of GFP driven by ECM promoters P_*bslA*_, P_*eps*_ and P_*tapA*_ (the promoter of the *tapA*-*sipW*-*tasA* operon) was clearly inhibited by the presence of VCs. On the other hand, the expression from P_*hag*_ appeared to be increased (Fig. 2a and Supplementary Figs. 4 and 5)—consistent with the mutually exclusive regulation of matrix production and flagellar motility^42^. We then used flow cytometry to quantify the level of expression of those reporters over time (Fig. 2b and Supplementary S6). VCs reduced the number of cells that express all ECM operons at all-time points measured, and increased the expression of *hag* at early stages of colony development (days 1 and 2).

Previous studies reported an antibacterial effect of VCs, which in some settings inhibit planktonic growth of bacteria^37^. To rule out the possibility that the defective phenotypes observed here were due to growth inhibition, we examined the effect of VCs on the growth of mutants lacking either the ECM genes (*Δeps*, *ΔtasA*)^43^ or their activator (*ΔsinI*)^44^. Those mutants’ growth rates in shaking culture are comparable or higher than WT^16^, but they form small and unstructured colonies and fail to develop into biofilms. As the 3D structure of a biofilm colony supports a larger bacterial population, all mutants that fail to form the correct colony architecture have lower biomass than the wild-type biofilms^45^. The growth of those featureless mutants was not affected by exposure to VCs, as judged by the number of cells in biofilms grown in the presence and the absence on VCs (Supplementary Fig. 7). On the other hand, the amount of EPS extracted from the same amount of cells of VCs-treated colonies was significantly reduced (Supplementary Table 3). This is the likely explanation for the lower CFU counts in colonies exposed to VCs (Fig. 1c) as they fail to produce EPS and do not form fully developed structures, they thus support a smaller population. Taken together, the results presented suggest that VCs produced by *B. subtilis* inhibit the development of neighbouring biofilm colonies by specific suppression of biofilm transcriptional programme.

### Volatiles are commonly used to inhibit competitors during the interspecies competition

**Fig. 3:**
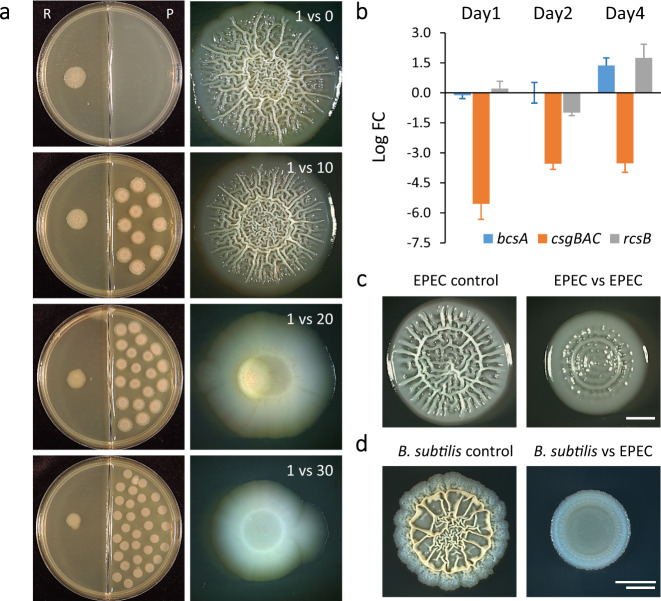
Volatiles can inhibit biofilm development across bacterial species. **a** Enteropathogenic *E. coli* biofilm colonies (R—receiver) grown on solid LBNS medium in the presence of an indicated number of neighbouring *B. subtilis* colonies (P—producer) grown on B4 medium. Left—experimental setting, right—a close-up of the receiving colony. Colonies were incubated for 2 days at 30 °C. Images are representative of (*n* > 3) independent experiments. **b** RT-PCR analysis of the expression levels of the indicated genes afters exposure to VCs. Presented are average fold-changes (FC) of expression in EPEC colonies (*n* = 3) grown either alone, or in the presence of 30 neighbouring *B. subtilis* colonies, for indicated times. Bars represent standard deviation. The experiment was repeated three times with similar results. **c** *E. coli* biofilm colonies grown on solid LBNS medium either alone (EPEC control) or in the presence of 30 neighbouring EPEC colonies. Colonies were incubated for 2 days at 30 °C. Images are representative of (*n* > 3) independent experiments. Scale bar 2 mm. **d** *B. subtilis* biofilm colonies grown on solid B4 medium either alone (*B. subtilis* control) or in the presence of 30 neighbouring EPEC colonies grown on LBNS medium. Colonies were incubated for 2 days at 30 °C. Images are representative of (*n* > 3) independent experiments. Scale bar 2 mm.

Volatiles are frequently used as a cross-species signalling molecule. EPEC is an enteropathogenic *E. coli* strain that can reside in contaminated soils, and therefore frequently shares the same niche as *B. subtilis*^46,47^. In our experimental setting, EPEC biofilm development could be inhibited by VCs produced by neighbouring *B. subtilis* colonies, in a dose-dependent manner (Fig. 3a). In this case, no morphological changes were observed below critical biomass for inhibition (20 colonies), but after crossing this threshold, the colonies that developed were flat and featureless. Just like in the case of *B. subtilis*, the defect in morphology was accompanied by a decrease in colony size and in the number of viable cells in the EPEC colony (Supplementary Fig. 8). In contrast to the self-inhibition of *B. subtilis*, the inhibition of EPEC was not relieved with time, and no 3D structure developed at any time point tested (Supplementary Fig. 9).

In *E. coli* biofilms, the main components of ECM are Curli (amyloid fibres encoded by the *csgB* operon) and the exopolysaccharide cellulose (encoded by *bcsA)*^48,49^. Under most conditions, Curli fibres are essential for establishing 3D morphology, while the role of exopolysaccharides is strain and condition dependent^49^. The RT-qPCR analysis revealed that the presence of VCs had little or no effect on *bcsA* expression, but dramatically reduced the expression of *csgB* (Fig. 3b). In contrast, the expression of the transcription factor *rcsB*^50^, regulating the biosynthesis of colanic acid (a negatively charged exopolysaccharide that forms a protective capsule^51^), was induced by VCs. These results suggest that VCs produced by *B. subtilis* serve as a specific cue inhibiting amyloid production by *E. coli* biofilms.

To determine whether the production of biofilm-inhibiting VCs is a common mechanism in bacterial competition, we next used EPEC as VCs producer. Consistent with the general nature of olfactory warfare in bacteria, VCs produced by EPEC could inhibit its own biofilm development (Fig. 3c), as well as the development of *B. subtilis* colonies (Fig. 3d).

### Self-produced organic volatiles, 3-methyl-1-butanol and 2-nonanon, confirm specific inhibition of biofilm development

*B. subtilis* produces a broad range of volatile compounds, with volatiles profiles differing dramatically between strains and growth conditions^52^. We first directly tested the effect of a central bacterial inorganic volatile—ammonia. We found that under our conditions (colonies grown on biofilm-inducing medium), 3 mL of 2% v/v ammonia was lethal. When applied at a lower concentration, ammonia interfered with biofilm formation, as judged by defects in colony structure (Supplementary Fig. 10), and increased biofilm spreading. The morphology defects on the rich B4 medium were more severe than on the defined MSgg (Supplementary Fig. 10, compare 0.02% v/v). In contrast to our findings, previous reports showed that ammonia induces floating biofilm formation in *B. subtilis* and *B. licheniformis*^37,38^. However, the same two studies reported contradictory effects of ammonia on *E. coli* biofilm formation, raising the possibility that its effects are highly context-dependent.

**Fig. 4:**
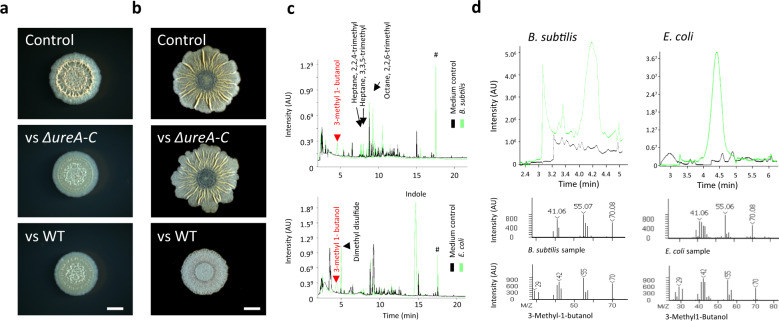
3-methyl-1-butanol is commonly produced by *B. subtilis* and *E. coli* biofilms to inhibit biofilm formation. **a** *B. subtilis* 3610 biofilm colonies grown on solid B4 medium either alone, or in the presence of 30 neighbouring colonies (either WT or Δ*ureA-C*, as indicated). Colonies were incubated for 2 days at 30 °C. Images are representative of (*n* > 3) independent experiments. **b** Experiment was performed as in **a** on MSgg medium. **c** Total ion chromatogram profiles of *B. subtilis* and EPEC volatiles produced by its biofilm as judged by GC–MS. Headspace analysis of a medium control is in black, and of a biofilm colony in green. Peaks were cross-referenced with National Institute of Standards and Technology (NIST) and Wiley libraries. **d** Representative Selected mass (55.07 Da) chromatogram of 3-methyl-butanol of the produced by *B. subtilis* (on MSgg) and *E. coli* (on LBNS). Headspace analysis of a medium control is in black, and of a biofilm colony in green. MS spectra of the standard (3-methyl-1-butanol) and peak identified as 3-methyl-1-butanol in *B. subtilis* (on MSgg) and *E. coli* (on LBNS) is provided. Experiments were performed with three independent repeats and four technical repeats.

To gain more insight into the role of ammonia in biofilm colony development, we used *B. subtilis* mutant lacking urease (Δ*ureA-C*), and thus unable to produce ammonia. When this mutant was used as a VCs producer, it was still able to efficiently inhibit neighbouring wild-type biofilm development on B4 medium, suggesting that self-produced ammonia plays no role in biofilm inhibition in this setting (Fig. 4a). On the other hand, on the defined MSgg medium, the inhibitory effect of the mutant was less pronounced, suggesting a more central role for ammonia (Fig. 4b). Taken together, those results suggest that while biofilms grown on B4 are more sensitive to inhibition by ammonia, only biofilms growing on MSgg use it to inhibit competing biofilms. An additional inorganic volatile, carbon dioxide, did not prevent the formation of robust wrinkles of exposed colonies (Supplementary Fig. 11).

**Fig. 5:**
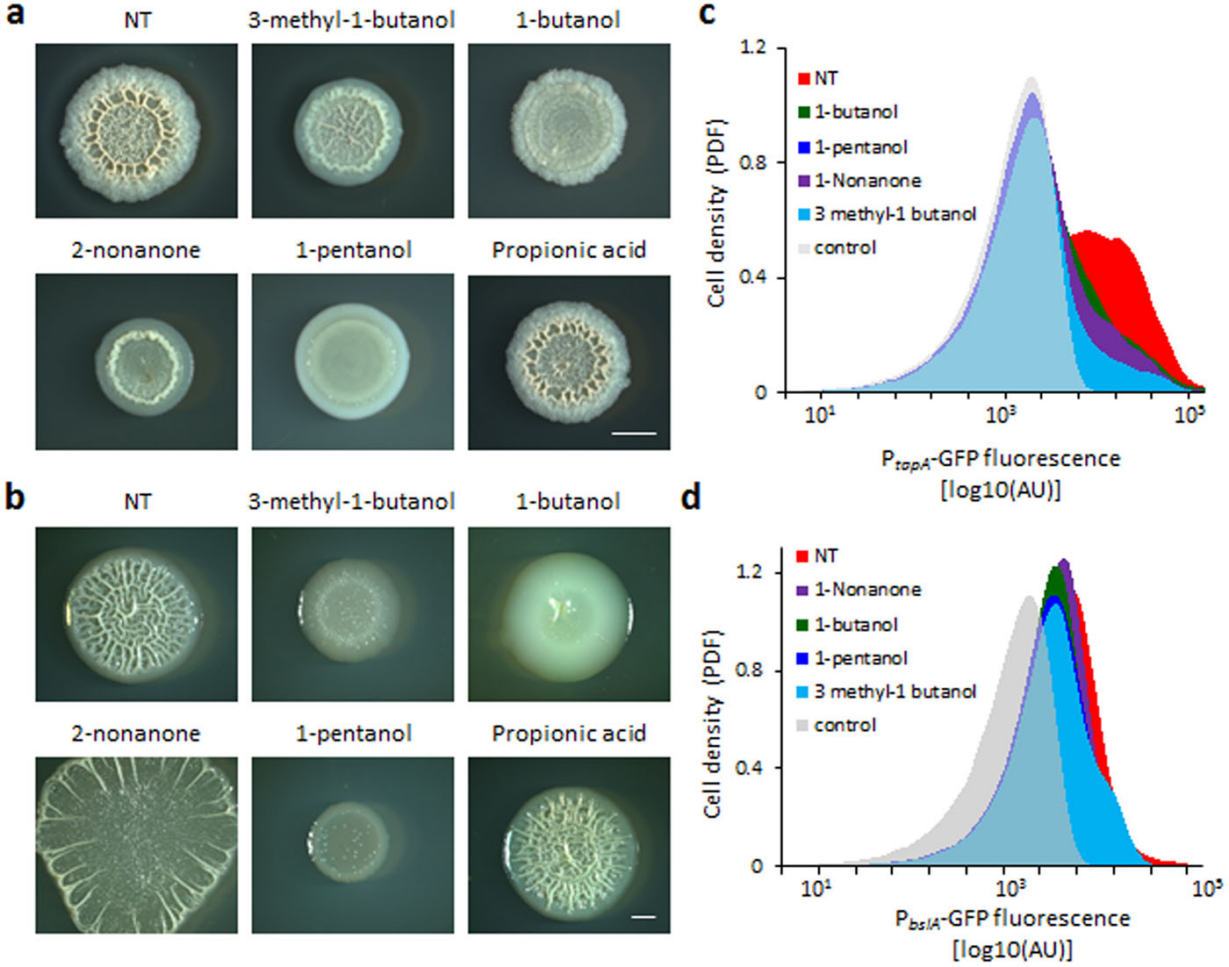
Structurally related organic VOCs inhibit biofilm formation and ECM expression. **a** *B. subtilis* 3610 biofilm colonies grown on solid B4 medium either alone (NT) or in the presence of the indicated volatiles. The commercial volatiles were added as 3 mL of 0.2% v/v solution placed in a divided agar-plate in Fig. 1b. Colonies were incubated for 2 days at 30 °C. Images are representative of (*n* > 3) independent experiments. Scale bar 2 mm. **b** *E. coli* biofilm colonies grown on solid LBNS medium either alone (NT) or in the presence of indicated commercial volatiles, added as in **a**. Colonies were incubated for 2 days at 30 °C. Images are representative of (*n* > 3) independent experiments. Scale bar 2 mm. **c** *B. subtilis* 3610 strains carrying P_tapA_-GFP or P_bslA_-GFP were incubated for 2 days either alone (NT) or in the presence of the indicated commercial volatiles. Control (grey)—autofluorescence levels of the parental non-fluorescent strain. Shown are representative results of three independent experiments performed with least two technical repeats.

The volatile repertoire produced by bacteria varies significantly depending on conditions^52^. To directly test which volatiles are produced by *B. subtilis* and *E. coli* biofilm colonies, we performed GC–MS of organic volatiles (VOCs). An untargeted approach identified a robust production of 3-methyl-1-butanol by both species (Fig. 4c), which was then verified by a targeted MS analysis against analytical standards (Fig. 4d). We used this targeted approach to evaluate the presence of several commercially available bioactive VOCs that are known to be produced by *B. subtilis*^35,52^ and were previously reported to modulate bacterial development. Those included propionic acid^39^; glyoxylic acid^41^; 2-nonanone^40^; 2-undercanone^40^; 1-butanol^37^; ethanol^37^; and 1-pentanol^41^. Gas chromatography-mass spectrometry (GC–MS) analysis revealed that under our conditions, *B. subtilis* biofilm colonies also produced 1-butanol and EPEC produced 2-nonanone (Supplementary Figs. 12 and 13). We next directly verified the effect of those VOCs on biofilm development, by adding them as a solution in a divided petri dish next to a single biofilm colony. All three VOCs identified by MS could inhibit the development of *B. subtilis* (Fig. 5a). 3-methyl-1-butanol and 1-butanol had a severe inhibitory effect on *E. coli* biofilm development, while the effect of 2-nonanone on was less pronounced (Fig. 5b). Out of the compounds not identified by MS, 1-pentanol (very similar in its structure to 1-butanol) had a strong inhibitory effect (Fig. 5a, b) and 2-undecanone was somewhat active, but only against *B. subtilis* (Supplementary Fig. 14). The rest of the compounds not identified in the biofilm headspace had no biofilm-inhibiting activity (Fig. 5a, b and Supplementary Fig. 14). Consistent with our finding that VCs inhibited biofilm development by reducing the expression of ECM operons, the commercial volatiles that inhibited biofilm morphology, also repressed the expression of *tapA-sipW-tasA, bslA* (Fig. 5c, d) and *eps* operons (Supplementary Fig. 15).

## Discussion

Interactions among the bacteria in the rhizosphere are intensely competitive, both within and between species. Fast-growing organisms compete for nutrients and space, constantly invading new niches. Many bacterial species defend their established niches by secreting antibiotics to prevent competitors from invading their territory^53^, however, antibiotics can only act in close proximity. We here describe an additional potential defence mechanism—bacteria producing VCs that prevent biofilm formation by invading bacteria.

The main effect of VCs was not direct growth inhibition or killing, as in the case of antibiotics. Instead, we found that specific inhibition of ECM production inhibited normal biofilm development and limited colony spreading. In biofilms, bacteria frequently migrate towards new niches by sliding, powered by cell division and ECM production^8,15,54^. Blocking this collective motility may serve as an effective strategy to distance competitors. Furthermore, preventing biofilm formation denies the potential invaders the fitness advantages associated with this life style^55^, such as better host attachment and phenotypic antibiotic resistance—making the invading bacteria more sensitive to the antibiotics present in the rhizosphere.

VCs-dependent inhibition was only evident when VCs producers reached certain critical biomass, and thus the inhibitory effect described here is by definition a feature of mature biofilms. One appealing ecological scenario is that once a critical mass of bacteria is achieved in a given location (pioneers), production of certain species of VCs will prevent the development of competing colonies in proximity, protecting this established community from potential competitors (newcomers). These findings expand the known range of VCs signalling during bacterial interactions; as while specific VCs served as weapons against invaders, other volatiles can promote cooperation between neighbour colonies at new colonisation sites^37,38^.

The role of bacterial VCs in inhibiting biofilm formation reveals an additional layer of the complex interactions in the competitive natural environments. A better understanding of the versatile roles of bacterial VCs can lead to the development of new strategies to control beneficial biofilm formation in environmental and agricultural settings.

## Methods

### Strains and media

The strains are summarised in Supplementary Table 1.

The media used were (i) B4 medium (0.4% yeast extract, 05% glucose, supplemented with calcium acetate as in refs. ^5,56^); (ii) MSgg, prepared as in ref. ^57^; (iii) modified MSgg, with phenyl alanine, tryptophan and threonine replaced by exudate collected from 35 day-old tomato plants (*Solanum lycopersicum*; cv. M82) cultivated in a hydroponic system under sterile conditions as described^58^; or (iv) in Luria–Bertani with no salt (LBNS) medium (0.5% yeast extract, 1% tryptone). All volatiles were purchased from Sigma-Aldrich with >98% purity.

Bacteria were grown at 30 °C. For all experiments, cultures were synchronised to OD_600_ = 0.2, and spotted on the appropriate solid growth media. All experiments were performed in a triplicate, with a minimum of four replicates for each condition.

### Viable cell quantification

Colonies were collected, resuspended in PBS (Biological Industries), and thoroughly vortexed. The samples were then mildly sonicated (BRANSON digital sonifier, Model 250, Microtip, amplitude 30%, pulse 2 × 5 s). To determine the number of colony-forming units (CFU), samples were serially diluted in PBS, plated on LB plates, and colonies were counted after incubation at 30 °C overnight.

### Imaging

All images were taken using a Nikon D3 camera or a Stereo Discovery V20″ microscope (Tochigi, Japan) with objectives Plan Apo S × 0.5 FWD 134 mm or Apo S × 1.0 FWD 60 mm (Zeiss, Goettingen, Germany) attached to a high-resolution microscopy Axiocam camera. Images were created and processed using Axiovision suite software (Zeiss).

### Flow cytometry

*B. subtilis* biofilms were inoculated as described above, and incubated for the time period indicated in the legend for each figure. Biofilms were then scraped from the plate surface and separated into single cells using mild sonication. Samples were fixated in 4% paraformaldehyde (Electron Microscopy Sciences) and kept at 4 °C until the measurement. Samples were analysed by LSR-II cytometer (Becton Dickinson, San Jose, CA, USA) operating a solid-state laser at 488 nm. GFP intensities were collected by 505 LP and 525/50 BP filters. For each sample, 10^6^ events were recorded and analysed for GFP intensities. The autofluorescence level was determined in each experiment by measuring a biofilm sample from a non-fluorescent strain of the same genetic background. The distribution of GFP intensities was analysed with using a custom Matlab code and visualised by Excel. The experiments were repeated three times, in technical duplicates, with similar results.

### Real-time PCR

EPEC colonies (*n* = 3) were collected, lysed in 250 µL lysozyme (20 mg mL^−1^) and incubated at 37 °C for 10 min. Next, 1 mL TRIzol Reagent (Bio-Lab, Israel) was added and RNA was extracted according to the manufacturer’s instructions. DNA contaminants were removed with TURBO DNA-free^TM^ kit (Invitrogen, USA), according to the manufacturer’s instructions.

cDNA synthesis was carried out by SuperScript® III First-Strand Synthesis System (Invitrogen, USA) according to the manufacturer’s instructions, from 200 ng total RNA using random hexamer primers. cDNA was then amplified with KAPA SYBR FAST qPCR Master Mix (2×) Universal (Sigma-Aldrich, USA), according to the manufacturer’s instructions. All primers used in this study (Supplementary Table 2) were purchased from Sigma-Aldrich. Real-time PCR was performed using the Applied Biosystems StepOnePlus Real-Time PCR Systems (Thermo Fisher Scientific, USA). The thermal cycle conditions were as follows: 10 min denaturation at 95 °C, followed by 40 cycles of amplification: 15 s at 95 °C and 60 s at 60 °C. For quantification, the CT of each gene was normalised to the CT of the housekeeping gene rrsG (Δ*C*_T_); and then the difference between treated and untreated samples was calculated (ΔΔ*C*_T_). Results are presented as fold-changes in expression (log2^−ΔΔCT^).

### VOCs analysis

For volatile collection, the colony biofilms were grown in collection vials on top of biofilm-inducing solid medium for 2 days at 30 °C. The headspace (300 mL) above cultures was actively sampled onto Tenax GR thermal desorption tubes at a flow rate of ~30 mL min^−1^ using a DHS module (Gerstel, Germany). Samples were collected under sterile conditions in a laminar flow hood. VCs analysis was conducted on a thermal desorption-gas chromatography time-of-flight mass spectrometer (GC-TOF-MS) platform (Leco BT, Germany) combined with Gerstel MPS autosampler (Germany). VOCs were desorbed from sorbent tubes using temperature gradient from 30 to 190 °C for 300 °C min^−1^, cryo-focused on a cold injection system (CIS, Gerstel, Germany) maintained at 2 °C and desorbed from the CIS onto the GC (Agilent 7890 A) by flash heating to 250 °C for 3 min. The GC column (DB-5MS column, 30 m, 0.25 mm internal diameter, 0.25 μm film thickness, Restek) was held at an initial temperature of 40 °C for 4 min, ramped to 200 °C at 10 °C min^−1^ and to 300 °C at 15 °C min^−1^ for 4 min. The GC runtime was 30 min with a total TD cycle time of 30 min. The TOF-MS was in electron ionisation mode set at 70 eV. The source temperature was set to 220 °C, and spectra were acquired in dynamic range extension mode at 20 scans s^−1^ over a range of 35–650 *m*/*z*.

#### Data processing

GC-TOF-MS data were acquired and analysed using ChromaTof (Leco, Germany). Chromatographic peaks and mass spectra were cross-referenced with National Institute of Standards and Technology (NIST17) and Wiley libraries for putative identification purposes (matching factor >800 match) and compared with retention time and spectra of injected of reference standards.

### Reporting summary

Further information on research design is available in the Nature Research Reporting Summary linked to this article.

## Supplementary information

Supporting Information

Reporting Summary

## Data Availability

All data generated or analysed during this study are included in this published article (and its supplementary information files).
