# Supplementary material for: Weaponizing volatiles to inhibit competitor biofilms from a distance
Source: NPJ Biofilms Microbiomes. 2021 Jan 5;7:2. doi: 10.1038/s41522-020-00174-4 (PMC7785731; doi:10.1038/s41522-020-00174-4)
Supplement: Supplementary file 1 — Supporting Information [file 41522_2020_174_MOESM1_ESM.pdf]

Title: Weaponizing Volatiles to Inhibit Competitor Biofilms from a Distance

Qihui Hou[1], Alona Keren-Paz[1], Elisa Korenblum[2], Rela Oved[1], Sergey Malitsky[3] and Ilana Kolodkin-Gal[1+]

Supplementary information

Content:

Supplementary Figures (1-15)

Supplementary Tables (1-3)

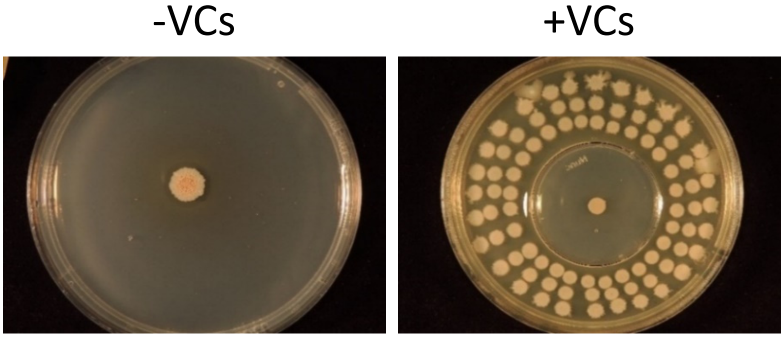

Supplementary Figure 1.

B. subtilis 3610 colony was inoculated on B4 agar, in the center of a 4-cm petri dish placed on top of a standard 9-cm petri dish. The larger plate contained B4 agar inoculated with 80 *B. subtilis* colonies. The plates were sealed and incubated for 4 days at 30ºC.

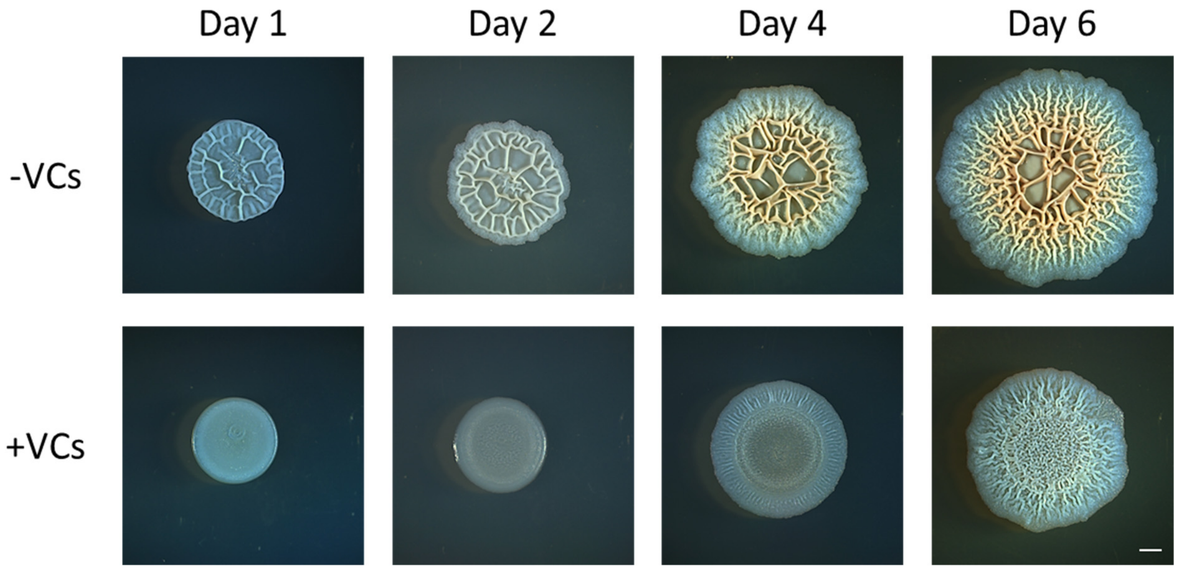

Supplementary Figure 2.

B. subtilis 3610 biofilm colonies grown on solid B4 medium in the presence of 20 neighboring colonies. Colonies were incubated at 30ºC for indicated times. Scale bar – 2 mm. Images are representative of (n>3) independent experiments.

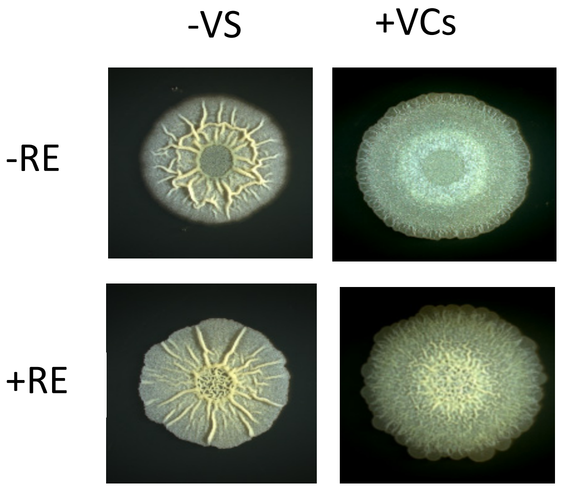

Supplementary Figure 3.

B. subtilis 3610 biofilm colonies grown on modified MSgg agar, with amino acids either completely removed (-RE) or replaced by root exudate (+RE), either alone (-VCs) or in the presence (+VCs) of 20 neighboring colonies. Colonies were incubated at 30ºC for 2 days. Scale bar – 2 mm. Images are representative of (n>3) independent experiments.

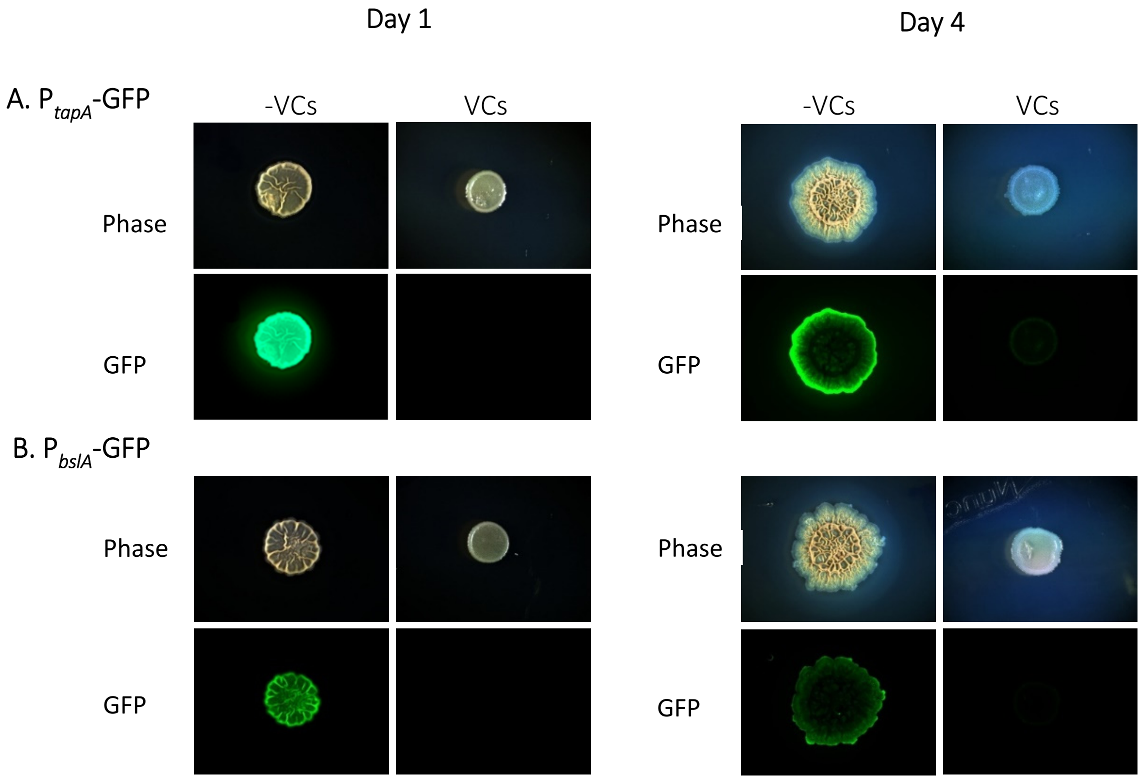

Supplementary Figure 4.

Top-down phase and fluorescent (GFP) images of *B. subtilis* 3610 strains carrying (**A**) P_{tapA}-GFP or (**B**) P_{bslA}-GFP were incubated either alone (-VCs) or in the presence of 20 volatile producers (+VCs). Colonies were inoculated on solid B4 medium, and incubated at 30ºC for the indicated amount of time. Images are representative of (n>3) independent experiments.

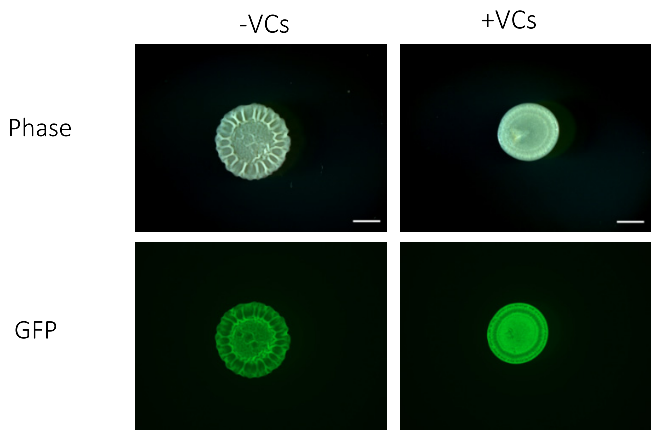

Supplementary Figure 5.

Top-down phase and fluorescent (GFP) images of *B. subtilis* 3610 strains carrying P_{hag}-GFP incubated either alone (-VCs), or in the presence of 20 volatile producers (+VCs). Colonies were inoculated on solid B4 medium, and incubated at 30ºC for 24 h. Scale bar – 2 mm. Images are representative of (n>3) independent experiments.

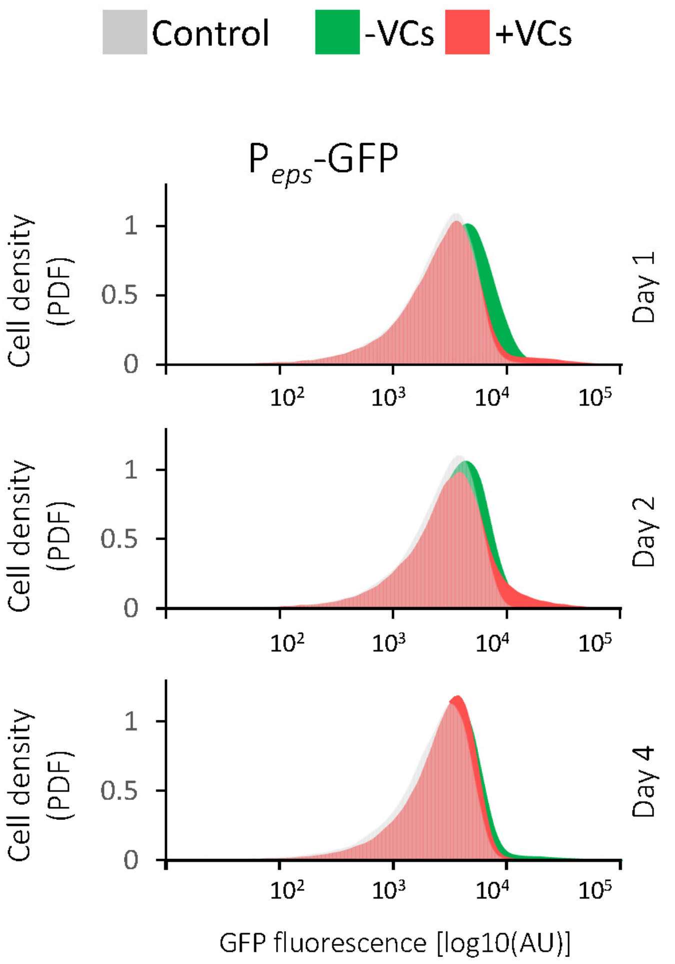

Supplementary Figure 6.

Flow cytometry analysis of *B. subtilis* 3610 strains carrying P_{eps}-GFP reporter incubated either alone (green, -VCs) or in the presence of 20 volatile producers (red, +VCs). Control (grey) – autofluorescence levels of the non-fluorescent parental strain. Colonies were grown on B4 agar, at 30°C for 1, 2 or 4 days, as indicated. Shown are representative results of three independent experiments performed with least two technical repeats.

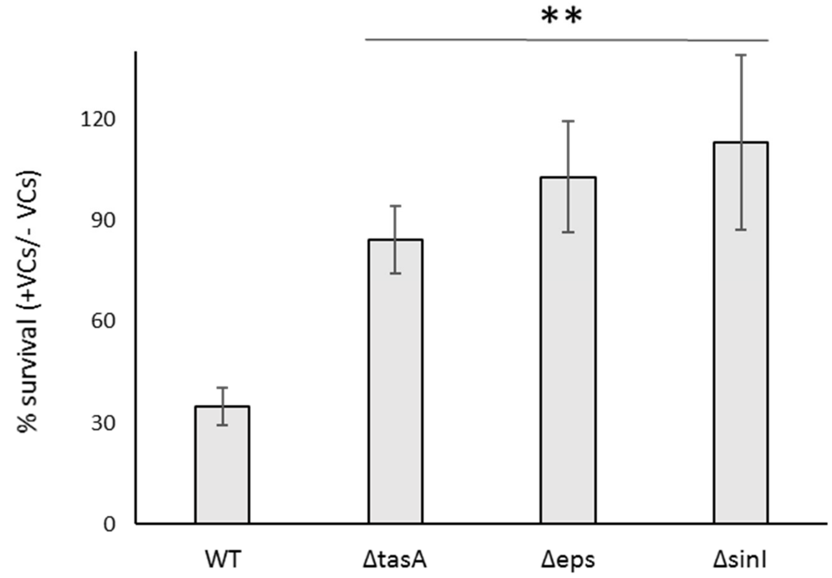

Supplementary Figure 7.

B. subtilis 3610 parental strain (WT) and the indicated deletion mutants, were grown on B4 agar, either alone (-VCs) or in the presence of 25 volatile producers (+VCs). Colonies (at least 6 for each strain and each condition) were incubate for 4 days at 30°C. CFU counts were determined, and the survival percentage was calculated. P values, as determined by ANOVA followed by TukeyHSD are indicated (** pVal <0.001 as compared to WT).

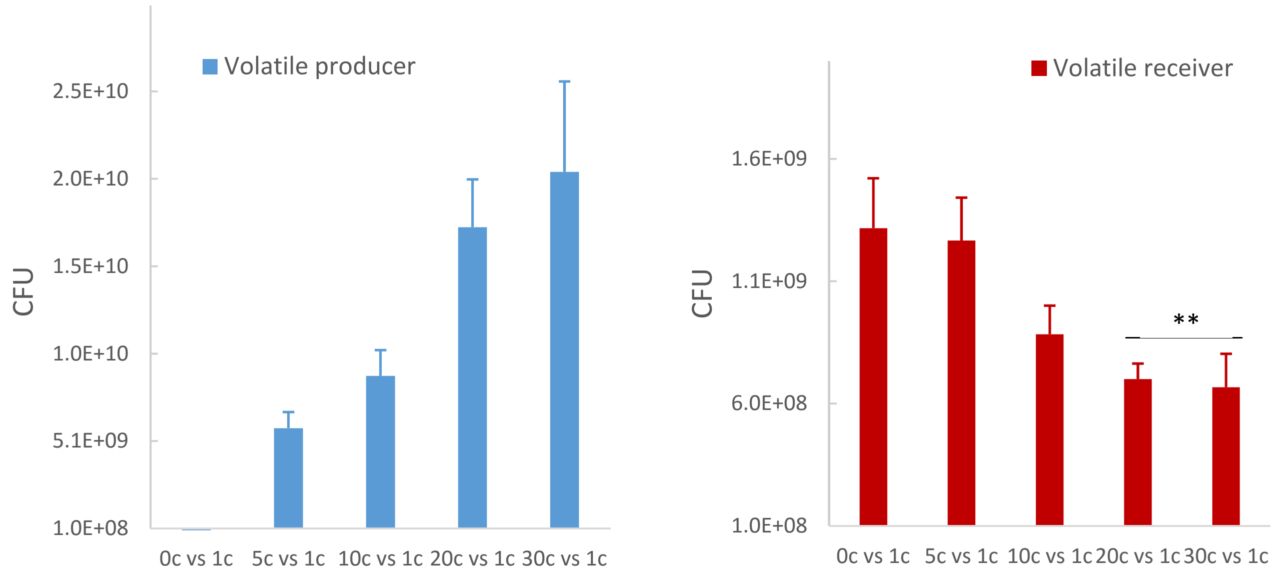

Supplementary Figure 8.

Colony forming units were determined for *B. subtilis* VCs producer (left, blue) and EPEC receiver (right, red). Colonies (n=6) were grown on biofilm on biofilm-inducing media (B4 for *B. subtilis* and for LBNS for EPEC) for 2 days before CFU was quantified. P values, as determined by ANOVA followed by TukeyHSD are indicated (** pVal <0.001 vs 0 VCs producers).

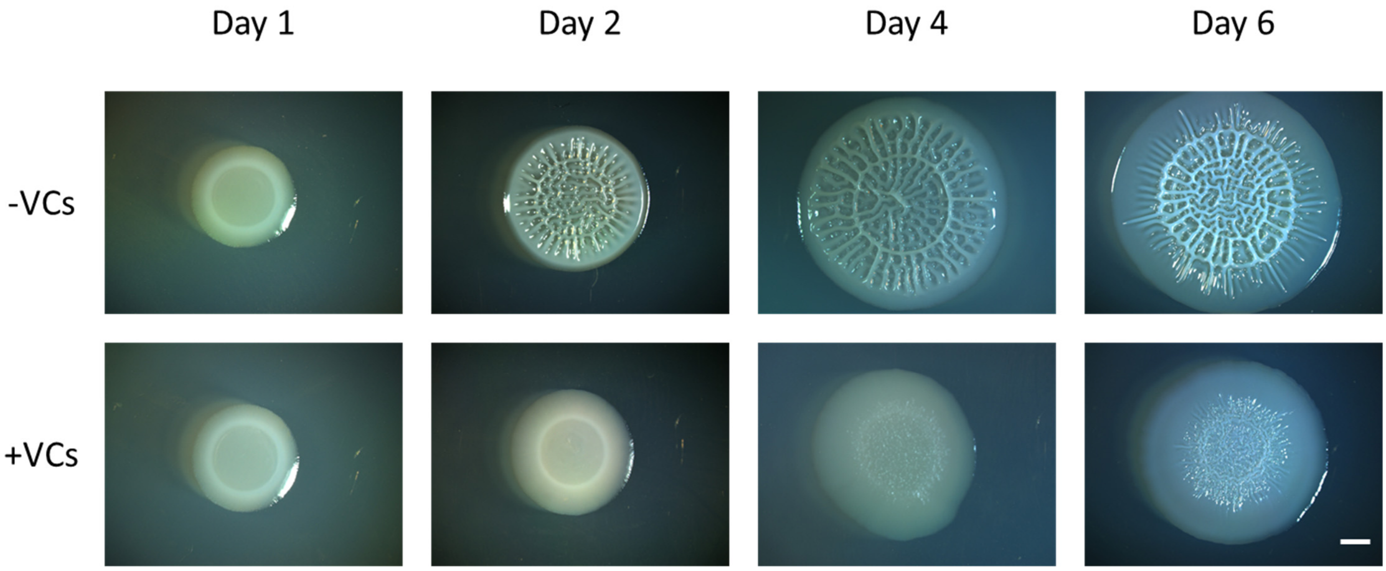

Supplementary Figure 9.

EPEC biofilm colonies grown on solid LBNS medium in the presence of 30 neighboring *B. subtilis* colonies. Colonies were incubated at 30ºC for indicated times. Scale bar – 2 mm. Images are representative of (n>3) independent experiments.

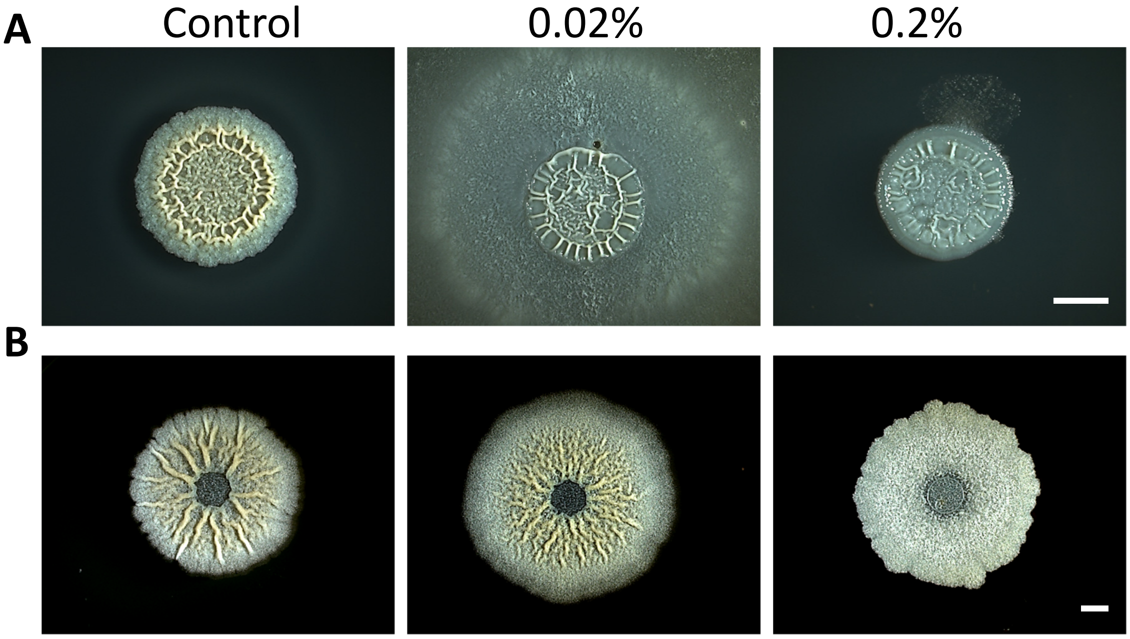

Supplementary Figure 10.

B. subtilis biofilm colonies grown on solid B4 (**A**) and MSgg (**B**) medium either alone (control) in the presence of 3 ml of ammonia at indicated v/v concentrations. Colonies were incubated at 30ºC for 2 days. Scale bar – 2 mm. Images are representative of (n>3) independent experiments.

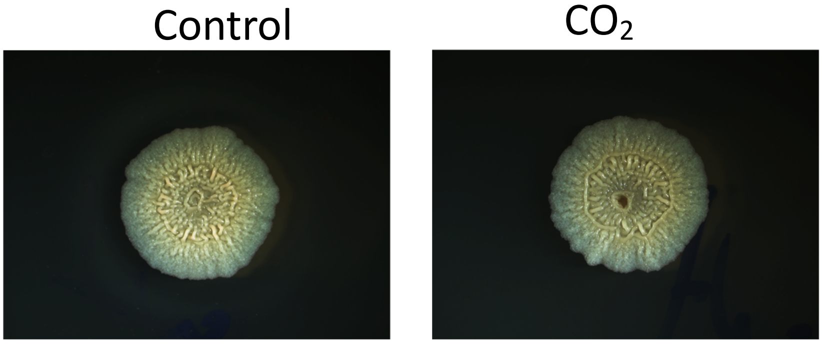

Supplementary Figure 11.

B. subtilis biofilm colonies grown on solid B4 medium either alone (control) in the presence of Carbon dioxide (GasPak CO2 container system). Colonies were incubated at 30ºC for 3 days. Scale bar – 2 mm. Images are representative of (n=3) independent experiments, performed with three technical repeats.

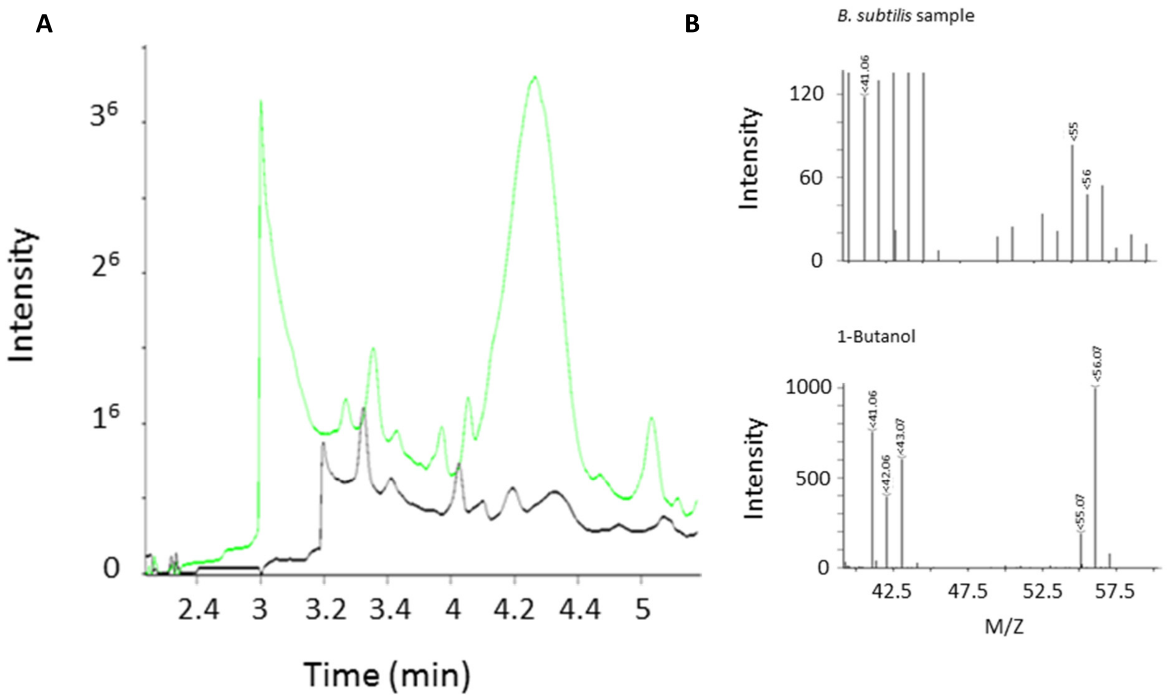

Supplementary Figure 12.

(A) Selected mass (56.07 Da) chromatogram of the headspace for MSgg (black) versus *B. subtilis* biofilms (green) for a compound eluted at 3.0 min and putatively identified as 1-butanol by comparison to analytical standard. (**B**) Mass spectrum of 1-butanol standard with (retention time 3.0 min) versus mass spectrum of the same peak from *B. subtilis* biofilms. Putatively identified 1-butanol peak in *B. subtilis* samples was co-eluted with additional compounds

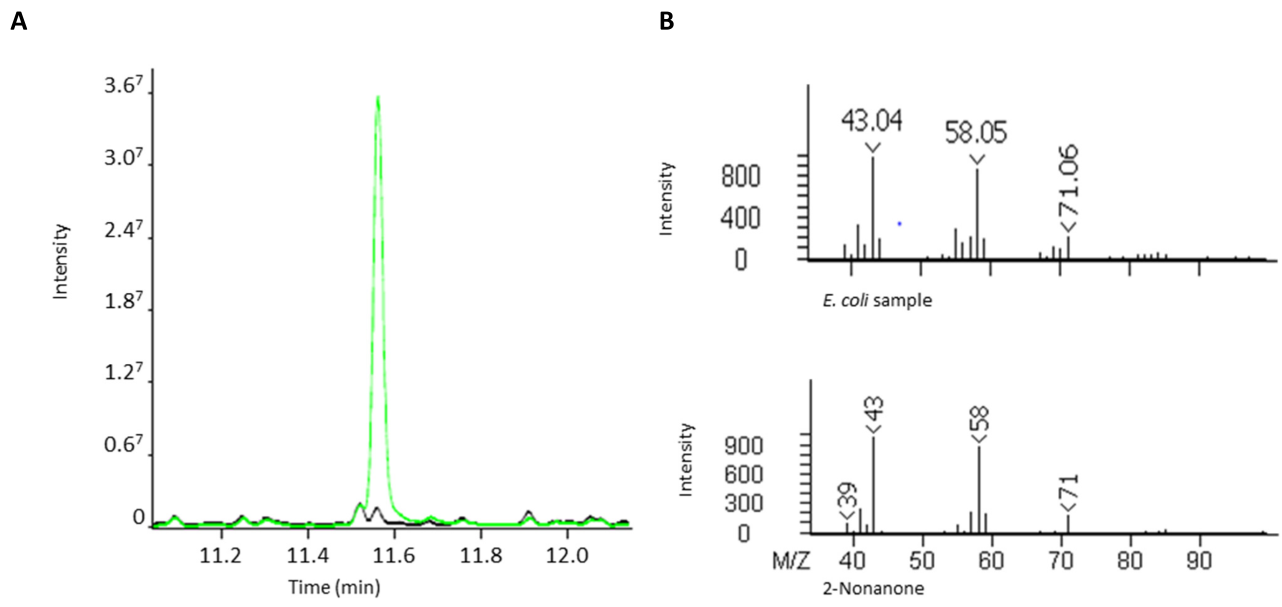

Supplementary Figure 13.

(**A**) Selected mass (58.05) chromatogram of the headspace for LBNS (black) versus *E. coli* biofilms (green) for a compound identified as 2-nonanone. (**B**) Mass spectrum of 2-nonanone standard versus mass spectrum of the same peak form *E. coli* chromatogram. 2-nonanone was identified by comparison to the analytical standard elution time and mass spectra.

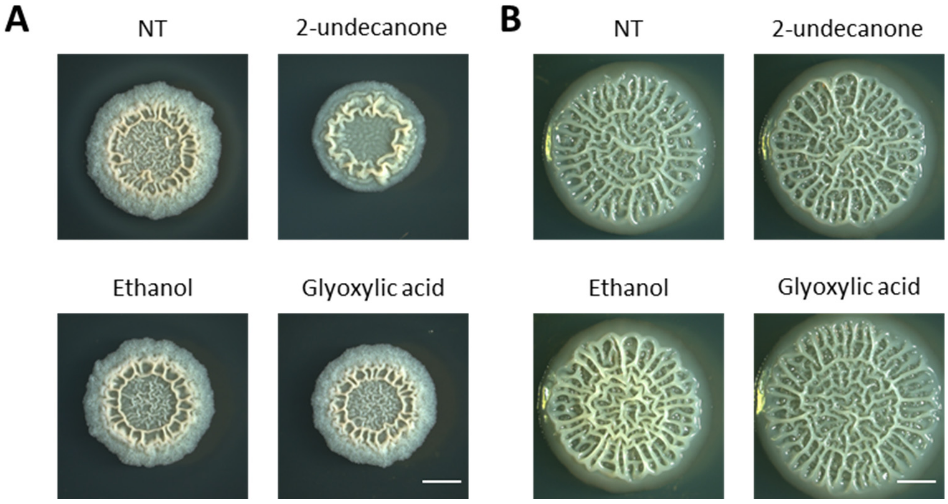

Supplementary Figure 14.

A. *B. subtilis* 3610 biofilm colonies grown on solid B4 medium either alone (NT) in the presence of the indicated volatiles. The volatiles were added as 3 ml of 2% v/v, placed in a divided agar-plate as in Figure 1B. Colonies were incubated for 2 days at 30ºC. Images are representative of (n>3) independent experiments**.** Scale bar – 2 mm. **B.** *E. coli* biofilm colonies grown on solid LBNS medium either alone (NT) or in the presence of the indicated volatiles. The volatiles were added as 3 ml of 2% v/v, placed in a divided agar-plate as in Figure 1B. Colonies were incubated for 2 days at 30ºC. Images are representative of (n>3) independent experiments. Scale bar – 2 mm.

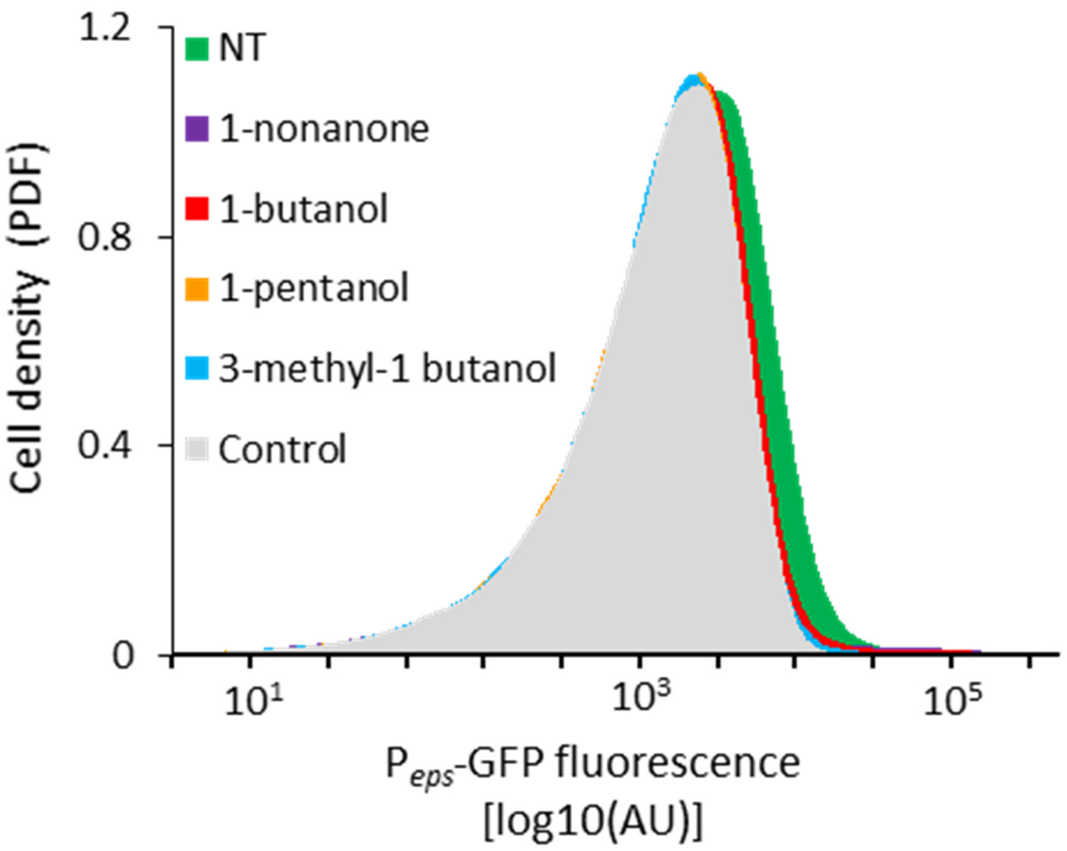

Supplementary Figure 15.

B. subtilis 3610 strains carrying P_{eps}-GFP or P were incubated either alone (NT) or in the presence of the indicated volatiles, added as in figure 5A. Shown are representative results for flow cytometry analysis out of three independent experiments performed with least two technical repeats.

Supplementary Table 1: Strains used in this study

No.	Genotype	Reference
Bacillus subtilis		
1	Wild-type (NCIB3610)	[1]
2	Δ*tasA*::*kan*	[2]
3	Δ*epsH*::*tet*	[2]
4	Δ*sinI*::*spec*	[2]
5	*amyE*::P_{hag}-*gfp*(*cam*)	[3]
6	*amyE*::P_{tapA}-*gfp*(*cam*)	[4]
7	*amyE*::P_{bslA}-*gfp*(*cam*)	This study, Constructed as in [5]
8	*amyE*::P_{eps}-*gfp*(*cam*)	[6]
Escherichia coli		
9	*Escherichia coli* E2348/69 (EPEC wild type isolate O127:H6)	[7], Kindly provided by Prof. Ilan Rosenshine (Hebrew University)

Supplementary Table 2: Primers used in this study

Name	Sequence(5’-3’)
bcsA-Forward	AAACGCGGTCAGCAGGAATA
bcsA-Reverse	ATCTTCCGGGTAGCTGTCCT
csgBAC-Forward	TGCCAGTATTTCGCAAGGTGC
csgBAC-Reverse	ACGTTGTGTCACGCGAATAGC
rcsB-Forward	GCCATTTCCCAAGCCTGTCG
rcsB -Reverse	CGGTCGGTGCACCTTGTTTC
rrsG-Forward	CTTGCTGCTTCGCTGACGAG
rrsG-Reverse	ATCCCATCTGGGCACATCCG

Supplementary Table 3: EPS (exopolymeric substances) reduction in VCs sensors

Strain	WT		*ΔepsΔtasA*	
Treatment	-VCs	+VCs	-VCs	+VCs
Average (mg)	0.675	0.25	<0.01	<0.01
STDEV	0.221736	0.129099	<0.01	<0.01

B. subtilis 3610 parental strain (WT) and the indicated EPS mutant, were grown on B4 agar, either alone (-VCs) or in the presence of 20 volatile producers (+VCs). Colonies were incubate for 3 days at 30°C. For each independent repeat five technical repeats were pulled together, and the same amount of cells was analyzed for each condition (as judged by optical density). EPS were harvested as described in [8] until the isopropanol extraction. Dry weight of the EPS was measured by analytical weight following the complete evaporation of isopropanol. Results are average and standard deviation of four independent repeats. WT: pVal <0.05 vs (–VCs versus +VCs)as determined by Students t-test.

Supplementary References

1 Branda, S. S., Gonzalez-Pastor, J. E., Ben-Yehuda, S., Losick, R. & Kolter, R. Fruiting body formation by Bacillus subtilis. *Proc Natl Acad Sci U S A* **98**, 11621-11626, doi:10.1073/pnas.191384198 (2001).

2 Branda, S. S., Chu, F., Kearns, D. B., Losick, R. & Kolter, R. A major protein component of the Bacillus subtilis biofilm matrix. *Mol Microbiol* **59**, 1229-1238, doi:10.1111/j.1365-2958.2005.05020.x (2006).

3 Kearns, D. B. & Losick, R. Cell population heterogeneity during growth of Bacillus subtilis. *Genes Dev* **19**, 3083-3094, doi:10.1101/gad.1373905 (2005).

4 Steinberg, N. *et al.* The extracellular matrix protein TasA is a developmental cue that maintains a motile subpopulation within Bacillus subtilis biofilms. *Sci Signal* **13**, doi:10.1126/scisignal.aaw8905 (2020).

5 Mhatre, E. *et al.* Presence of Calcium Lowers the Expansion of Bacillus subtilis Colony Biofilms. *Microorganisms* **5**, doi:10.3390/microorganisms5010007 (2017).

6 Chai, Y., Chu, F., Kolter, R. & Losick, R. Bistability and biofilm formation in Bacillus subtilis. *Mol Microbiol* **67**, 254-263, doi:10.1111/j.1365-2958.2007.06040.x (2008).

7 Pal, R. R. *et al.* Pathogenic E. coli Extracts Nutrients from Infected Host Cells Utilizing Injectisome Components. *Cell* **177**, 683-696 e618, doi:10.1016/j.cell.2019.02.022 (2019).

8 Ganin, H. *et al.* Indole derivatives maintain the status quo between beneficial biofilms and their plant hosts. *Mol Plant Microbe Interact*, doi:10.1094/MPMI-12-18-0327-R (2019).
