# Supplementary material for: Weaponizing volatiles to inhibit competitor biofilms from a distance
Source: NPJ Biofilms Microbiomes. 2021 Jan 5;7:2. doi: 10.1038/s41522-020-00174-4 (PMC7785731; doi:10.1038/s41522-020-00174-4)
Supplement: Supplementary file 2 — Reporting Summary [file 41522_2020_174_MOESM2_ESM.pdf]

nature research

Corresponding author(s): IKG
Last updated by author(s): 13102020

Reporting Summary

Nature Research wishes to improve the reproducibility of the work that we publish. This form provides structure for consistency and transparency in reporting. For further information on Nature Research policies, see our Editorial Policies and the Editorial Policy Checklist.

Statistics

For all statistical analyses, confirm that the following items are present in the figure legend, table legend, main text, or Methods section.

n/a	Confirmed	
☐	☒	The exact sample size (n) for each experimental group/condition, given as a discrete number and unit of measurement
☐	☒	A statement on whether measurements were taken from distinct samples or whether the same sample was measured repeatedly
☐	☒	The statistical test(s) used AND whether they are one- or two-sided<br>*Only common tests should be described solely by name; describe more complex techniques in the Methods section.*
☐	☒	A description of all covariates tested
☐	☒	A description of any assumptions or corrections, such as tests of normality and adjustment for multiple comparisons
☐	☒	A full description of the statistical parameters including central tendency (e.g. means) or other basic estimates (e.g. regression coefficient) AND variation (e.g. standard deviation) or associated estimates of uncertainty (e.g. confidence intervals)
☐	☒	For null hypothesis testing, the test statistic (e.g. F, t, r) with confidence intervals, effect sizes, degrees of freedom and P value noted<br>*Give P values as exact values whenever suitable.*
☒	☐	For Bayesian analysis, information on the choice of priors and Markov chain Monte Carlo settings
☒	☐	For hierarchical and complex designs, identification of the appropriate level for tests and full reporting of outcomes
☒	☐	Estimates of effect sizes (e.g. Cohen's d, Pearson's r), indicating how they were calculated

Our web collection on statistics for biologists contains articles on many of the points above.

Software and code

Policy information about availability of computer code

Data collection	NA
Data analysis	NA

For manuscripts utilizing custom algorithms or software that are central to the research but not yet described in published literature, software must be made available to editors and reviewers. We strongly encourage code deposition in a community repository (e.g. GitHub). See the Nature Research guidelines for submitting code & software for further information.

Data

Policy information about availability of data

All manuscripts must include a data availability statement. This statement should provide the following information, where applicable:

- Accession codes, unique identifiers, or web links for publicly available datasets
- A list of figures that have associated raw data
- A description of any restrictions on data availability

Provide your data availability statement here.

Field-specific reporting

Please select the one below that is the best fit for your research. If you are not sure, read the appropriate sections before making your selection.

☒ Life sciences ☐ Behavioural & social sciences ☐ Ecological, evolutionary & environmental sciences

For a reference copy of the document with all sections, see nature.com/documents/nr-reporting-summary-flat.pdf

Life sciences study design

All studies must disclose on these points even when the disclosure is negative.

Sample size	Sample size for each experiment is indicated within each legend
Data exclusions	No data was excluded
Replication	reproducibility is indicated for each experiment
Randomization	Samples were allocated into groups based on the indicated treatments
Blinding	Blinding was not feasible as most experiments were assigned by a single post doctoral fellow, However, they were reproduced independently and by using blinding with a lab technician

Reporting for specific materials, systems and methods

We require information from authors about some types of materials, experimental systems and methods used in many studies. Here, indicate whether each material, system or method listed is relevant to your study. If you are not sure if a list item applies to your research, read the appropriate section before selecting a response.

Materials & experimental systems

n/a	Involved in the study
☒	☐ Antibodies
☒	☐ Eukaryotic cell lines
☒	☐ Palaeontology and archaeology
☒	☐ Animals and other organisms
☒	☐ Human research participants
☒	☐ Clinical data
☒	☐ Dual use research of concern

Methods

n/a	Involved in the study
☒	☐ ChIP-seq
☐	☒ Flow cytometry
☒	☐ MRI-based neuroimaging

Flow Cytometry

Plots

Confirm that:

☒ The axis labels state the marker and fluorochrome used (e.g. CD4-FITC).

☒ The axis scales are clearly visible. Include numbers along axes only for bottom left plot of group (a 'group' is an analysis of identical markers).

☒ All plots are contour plots with outliers or pseudocolor plots.

☒ A numerical value for number of cells or percentage (with statistics) is provided.

Methodology

Sample preparation	B. subtilis biofilms were inoculated as described above, and incubated for the time period indicated in the legend for each figure. Biofilms were then scraped from the plate surface and separated into single cells using mild sonication, as previously described (Bloom-Ackermann et al 2016, Lopez et al 2009). Samples were fixated in 4% paraformaldehyde (Electron Microscopy Sciences) and kept at 4˚C until the measurement. Samples were analyzed by LSR-II cytometer (Becton Dickinson, San Jose, CA, USA) operating a solid-state laser at 488 nm. GFP intensities were collected by 505 LP and 525/50 BP filters. For each sample, 106 events were recorded and analyzed for GFP intensities. The auto-fluorescence level was determined in each experiment by measuring a biofilm sample from a non-fluorescent strain of the same genetic background. The distribution of GFP intensities was analyzed with using a custom Matlab code and visualized by Excel. The experiments was repeated 3 times, in technical duplicates, with similar results.

Instrument	LSR-II cytometer (Becton Dickinson, San Jose, CA, USA)
Software	Matlab
Cell population abundance	Indicated above
Gating strategy	Indicated above

☒ Tick this box to confirm that a figure exemplifying the gating strategy is provided in the Supplementary Information.
